# “At first, I was very afraid”—a qualitative description of participants’ views and experiences in the first Human Infection Study in Malawi

**DOI:** 10.12688/wellcomeopenres.16587.2

**Published:** 2021-10-04

**Authors:** Neema Mtunthama Toto, Kate Gooding, Blessings M. Kapumba, Kondwani Jambo, Jamie Rylance, Sarah Burr, Ben Morton, Stephen B. Gordon, Lucinda Manda-Taylor

**Affiliations:** 1 Malawi-Liverpool Wellcome Trust Clinical Research Programme, Blantyre, +265, Malawi; 2 Oxford Policy Management, Oxford, OX1 3HJ, UK; 3 Clinical Services, Liverpool School of Tropical Medicine, Liverpool, L3 5QA, UK; 4 University of Malawi College of Medicine, Blantyre, +265, Malawi

**Keywords:** Human infection studies, ethics, acceptability, Malawi

## Abstract

*Background: *Human infection studies (HIS) involve deliberately infecting healthy volunteers with a pathogen in a controlled environment to understand infection and support the development of effective vaccines or treatments. HIS research is expanding to many low and middle-income settings to accelerate vaccine development. Given the implementation of the first HIS research to establish the experimental human pneumococcal carriage model’s feasibility, we sought to understand the participant’s opinions and experiences.

*Methods:* We used a qualitative, descriptive approach to understand participants perceptions and experiences on HIS participation. Sixteen healthy adult participants were invited to participate in in-depth exit interviews to discuss their experiences, motivations and concerns.

*Results:* Our findings showed that the likelihood of participation in HIS research rests on three essential conditions: motivation to participate, compensation and advocacy. The motivation and decision to participate was based on reasons including altruism, patriotism, monetary and material incentives, and while compensation was deemed appropriate, concerns about unanticipated research-related risks were raised. Participant advocate groups were recommended for increasing awareness and educating others in the broader community about HIS research.

*Conclusions:* Participants’ experiences of HIS in Malawi provide the basis of what can be acceptable in HIS research in lower-income countries and areas where study procedures could be adjusted.

## Abbreviations

Community Advisory Groups                                  CAGs

Controlled Human Infection Studies                         CHIM

Experimental human pneumococcal carriage              EHPC

Grace Bandawe Conference Centre                          GBCC

Human infection studies                                          HIS

Liverpool School of Tropical Medicine                     LSTM

Low-middle income countries                                  LMICs

Malawi-Liverpool Wellcome Trust                           MLW

Malawi Accelerated Research in Vaccines using

Experimental and Laboratory Systems                       MARVELS

Malawi School Certificate of Education                      MSCE

National Health Sciences Research Committee           NHSRC

Participant Advisory Groups                                     PAGs

Polytechnic Students Union                                      PSU

Queen Elizabeth Central Hospital                              QECH

## Introduction

Human infection studies (HIS) or controlled human infection models (CHIM) often involve deliberately infecting healthy adult student volunteers with a pathogen in a controlled environment to better understand infection and support the development of effective vaccines or treatments ^1^. CHIM have been conducted in different participant groups over hundreds of years and have contributed vital scientific knowledge leading to advances in the development of drugs and vaccines. Recently, CHIM have received renewed interest, particularly in disease-endemic settings, because they offer an efficient and cost-effective approach for selecting the most promising vaccines for further development in populations that bear the more significant burden of infection ^2^. CHIM allows efficacy data to be generated quickly and promotes the identification of good immune correlations, the down-selection of vaccine candidates and early vaccine formulation decisions, and therefore provide an opportunity to circumvent the large-scale field efficacy studies to deselect intervention candidates ^2, 3^.

CHIM have been conducted in low and middle-income countries (LMICs) such as Columbia, Kenya, Tanzania and Thailand ^4^, Gabon ^5^, Equatorial Guinea, and Mali ^6^. Zambian scientists have conducted a pilot using a live attenuated Rotavirus vaccine in Zambia (Chilengi, 2020) ^7^. Plans to conduct CHIM in other settings such as India ^8^, Vietnam ^9^, Uganda ^10^, Malawi ^11^, to name a few, are underway to explore the acceleration of vaccine development relevant to LMIC populations. While CHIM offer scientific opportunities, there are some ethical issues concerning CHIM, especially in LMICs. Some authors raise concerns around benefit-sharing, limits to risk, the right to withdraw, informed consent, compensation for participants, and research with children or other vulnerable participants, for example, pregnant women ^12^. Other concerns relate to compensation, particularly if participant exposure to risks or harm is perceived as high, and potential burdens of participation are perceived to be impactful ^13^. Yet other concerns relate to issues of governance of CHIM research, particularly in LMICs. The lack of specific ethical frameworks and guidelines for ethics committees raises issues about a lack of review procedures that will guide the evaluation of human infection research ^12^. Other authors argue that these concerns are not unique to LMICs. Although the ethical issues may be more amplified in LMICs, they do not differ significantly from the ethical issues raised by CHIM in high-income countries ^14^. Despite the concerns, CHIM offers potential opportunities to quickly identify and develop vaccines and provide them to those who need them.

Evidence on experiences and decision making among CHIM participants remains limited, but a small number of articles have pointed to a range of motivations for taking part. Research with malaria CHIM participants in Kenya found that monetary compensation was a primary motivation ^6^. In the US, alongside financial incentives, participants reported altruism, and experiential motivations, such as curiosity, as reasons for participating in CHIM research ^15^. Beyond CHIM, there is limited evidence on motivation among healthy research volunteers. Only a few studies have examined why they join research, how they evaluate risks, and how well they understand these risks ^16^. A review of the literature on motivation among healthy volunteers across a range of clinical research studies (though almost all in high-income countries) found that participation was motivated by financial reward, contributing to science or the health of others, accessing ancillary healthcare benefits, interest in the science or goals of the study, meeting people and curiosity ^17^. Exploring participants’ perceptions and experiences in LMICs is essential for understanding what is appropriate, acceptable, and practically feasible to provide ethical guidance for the conduct of CHIM research.

## CHIM in Malawi

The Malawi-Liverpool Wellcome Trust Clinical Research Programme (MLW) has recently completed a feasibility study of experimental human pneumococcal carriage (EHPC) in Malawi ^18^ under an umbrella programme titled: Malawi Accelerated Research in Vaccines using Experimental and Laboratory Systems (MARVELS). MARVELS is a clinical research programme conducting CHIM for vaccine development – targeting both transmission and infection. The current research question that the MARVELS group of researchers seek to address is why pneumococcal conjugate vaccination in Malawi does not result in the decrease in vaccine-type pneumococcal carriage seen in other parts of the world. This question is highly tractable to the EHPC model. Before implementing the feasibility study, the MARVELS group conducted a formative qualitative study interviewing research staff, clinicians, district health officials, ethics committee members, medical students, and community representatives from rural and urban Blantyre to gather views on the acceptability of CHIM in Malawi. The study’s findings revealed that acceptability depended on various factors related to informed consent procedures, inclusion criteria, medical care and support, compensation, regulation and robust community engagement. The interviewed stakeholders also expressed concerns about the safety of study volunteers and distrust or confrontation from community members if a participant were to become ill ^19^. The findings informed the implementation of the pneumococcal feasibility study. In particular, information on stakeholder views helped MARVELS researchers understand how to design the study so that recruitment procedures addressed stakeholder concerns yet still maintained the scientific procedures based on an established pneumococcal carriage model from the Liverpool School of Tropical Medicine (LSTM), UK ^20^.

## Aim of the study

Building on the formative qualitative study of stakeholder views, this study aimed to describe the experiences, motivations and concerns of the enrolled healthy adult volunteers who had completed the MARVELS feasibility study. Specifically, we wanted to explore their opinions on study recruitment and consent procedures, medical care and support, compensation, and community engagement. We needed feedback from participants to help establish some degree of acceptability, identify areas where CHIM researchers in Malawi could improve the study design to ensure participants have a positive experience, minimise risk perceptions, and maximise their comfort. These findings will help inform the design of a pneumococcal conjugate vaccine (PCV13) trial in Malawi similar to that carried out in the UK ^20^.

## Methods

### Overview of the MARVELS pneumococcal feasibility study

In the MARVELS pneumococcal feasibility study, 24 healthy adult student volunteers were inoculated with viable *Streptococcus pneumoniae* serotype 6B or 0.9% saline (sham inoculation) to the inside of each nostril. Blood, throat swabs, saliva, nasal scrapes, and nasal wash samples were obtained at days 2, 7, and 14 post-inoculation following the pneumococcal challenge and participants were discharged from the study on day 21. Of the 24 participants enrolled in the study, some participants received a dose between 20,000 or 80,000 bacterial colony-forming units to each nostril (naris) in 100 µl of saline. A pre-specified randomisation list determined the randomisation of participants. Participants were monitored for safety and the establishment of the pneumococcal carriage during their enrollment. Participants were provided with a thermometer and antibiotics following inoculation. They were advised to monitor their temperature daily and report any signs and symptoms during the enrollment period. Any reported symptoms were characterised as mild, moderate or severe by the study doctor and treated according to a standard operating procedure. Participants were required to contact (text message/phone) a specified member of the research team before 12:00 hours every day for seven days post-inoculation irrespective of whether they had experienced symptoms or not. Participants were provided with hotel accommodation for the first three nights. A field nurse was stationed at the hotel during all participant overnight stays to monitor any adverse reactions that may have occurred within 24 hours of receiving the inoculum. Participants were then checked out from the hotel after their stay and reminded to return to the clinic on the scheduled visits. Participants received MWK 8,400 (~11 USD) per study visit as reimbursement for out-of-pocket expenses such as travel and compensating for time spent and burdens incurred while participating in the study. In total, participants received MWK 67,200 (~91 USD). The compensation offered in this study was consistent with the remuneration guidelines published in Malawi ^21^, paid pro-rata (per activity and not dependent on the completion of the study).

### Recruitment

Before recruitment, enrolment and consent procedures for the feasibility study, the MLW Science Communication department organised the public engagement activity at the University of Malawi’s Polytechnic college through the Polytechnic dean of students office and the student’s union (PSU). The Polytechnic campus is approximately 3km from QECH. After the public engagement activity, interested participants were given contact information and invited to a one-on-one visit to the research clinic. There they were briefed about the research and study procedures, including their participation in an exit interview. They were provided with an opportunity to ask questions and seek further clarifications. During this information visit, the research team did not ask participants to consent to the interview on the spot to give them a cooling-off period to make a decision, and subsequent non-engagement was taken as a decision not to participate. These two processes (the public engagement activity and the one-on-one research clinic informational visit) allowed participants to meet the research team, ask questions and have time for contemplation and reflection. These processes were designed to improve their understanding of the research and help their decision-making. Only on the day of screening and enrolment was consent for participation obtained ^18^.

### Study site

Interviews for the qualitative study were conducted in a private meeting room in the MLW research institution building. MLW is based in Blantyre and is situated adjacent to Queen Elizabeth Central Hospital (QECH). QECH is the largest government referral hospital in the country, with an official bed capacity of 1350. The hospital serves as the College of Medicine’s teaching and research hospital.

On their last scheduled study visit (day 21), we invited participants to participate in an exit interview. Study participants consented to participate in the exit interviews in the MLW research institution building. The sample size was not defined because the MARVELS pneumococcal feasibility study was powered to recruit only 36 participants to establish carriage. By the time we experienced our first wave of COVID-19 in April 2020, a total of 24 participants had completed the study. At that point, we interviewed 16 participants and observed through ongoing data analysis that no new information was being gathered or learned. At which point, we determined to have achieved saturation.

Biases within qualitative research are well-known, and although the exit interview was held on MLW premises, specific attention was given to avoid biases. Particular, attention was given to address interviewee biases knowing that interviewees may choose to withhold detailed descriptions or embellish them, mainly if the ‘truth’ is inconsistent with their preferred self-image, experience, opinion or wish to impress the interviewer ^22^. To address this concern, the interviewers made sure to probe, seek clarification, and continually refer to what the participant had said, mainly if there appeared to be contradictions. Attention was also given to reflexivity threats because while researchers wish to adopt a relatively neutral role, they may inadvertently demonstrate a preference for a particular perspective, and in the process, bias their findings ^22^. To this end, the two main social science researchers (NT and LMT) worked independently from the clinical team with a clear objective for reporting on the participants’ ethical concerns and experiences and continually discussed and agreed on codes, categories, and themes.

### Interviews

We used a semi-structured exit interview-guide that was piloted and tested in March 2019. The interview-guide ( Table 1) was piloted on two health workers to check for clarity, relevancy, comprehensiveness, and question flow. Questions that we identified as ambiguous were amended. Two social science researchers (NT and LMT) from the MARVELS project conducted exit interviews together or separately. Most interviews were conducted primarily in English, with one conducted in Chichewa, based on participant preferences. All interviews were conducted face-to-face. Interviews lasted about 60 minutes each, and the open-ended questions covered topics about reasons and decisions to participate, understanding of the purpose of the study, procedures and risks, views of information provision, satisfaction with study experiences, compensation and any social impacts on participation, for example, education, family or home environment. The semi-structured interview topics were developed based on common concerns about human challenge studies raised in ethics literature and the formative research by Kapumba *et al.* ^19^.

**Table 1.** Participants Exit Interviews Topic Guide.

	Question	Probe on the following
General views on the study	How did you hear about this study?	Motivation to participation?
	What made you decide to take part in this study?	
	What are your general experiences of participating in this kind of study	Anything that made you unsure about joining the study?
	How does that compare to how you feel about it now?	
Recruitment	What do you think about our study participant recruitment approaches?	Access to target population, use of flyers?
	What did you find the most important part of the recruitment process?	Participant information sheet? Opportunity to provide further details?
	What other recruitment approaches need to be considered for similar studies in the future?	
Screening and consenting	What can you tell me about your experiences with the screening procedures which included checking your eligibility to participate in the study?	Sample collection procedures, screening questions, and HIV testing?
	What was your experience with nasal washing and with nasal scraping?	Preference and why?
Pneumococci Inoculation	What can you tell me about your experiences with the procedures of being infected with the pneumococcal bacteria?	General feeling, fears, anticipated and unanticipated symptoms and AEs, disease expectations, and laboratory results for the collected samples?
	Did you have any thoughts on the risks of possible unexpected and (unconsented harm) from being infected with pneumococcal bacteria?	Harm to self, including possible harms to others and the environment?
Safety monitoring	What symptoms did you experience during the study period?	
	What is your general overview of the safety monitoring processes put in place?	Whether the safety monitoring procedures put in place satisfactory?
	Which approach worked for monitoring your safety? Calling and or SMS systems?	
	Was it enough? What need to be considered for future CHIM?	
	Follow-up visits – how convenient were they to you?	Time, frequency of the visits in days, facilities, travel, sample collection procedures?
	Were you given antibiotics to take at home? How important was it for you to have antibiotics at home? What need to be considered on the issue of antibiotics from your experience?	Information given on when to take the antibiotics?
Residential stay	What do you think about your experience of staying at Grace Bandawe for 3 days during the study?	Concerns, challenges, family or relations, work, or school demands? Effect on daily life experiences?
	What if you stayed at home? Would you consider that important?	
	What would need to be considered to ensure the residential stay is okay for future CHIM studies in Malawi?	Accommodation, food, time?
Compensation	What do you think about the money you received for being in this study? Does it seem like a fair amount? If yes, what makes it a fair amount? If no, what do you think would be a fair amount? What would make that amount fair?	Satisfactory, not enough, too much?
	What else do you think need to be considered on participant compensation?	
	What other means of compensation does the research team need to consider including for future human infection challenge studies?	
Final thoughts	What impact did participating in this study have on the quality of your life?	
	What are your unmet needs and opportunities of participating in this study?	
	Would you encourage others to participate in this kind of study?	
	Is there anything you feel could have been done better in this study?	Improvements in future CHIM studies?

### Data analysis procedures

Interviews were audio-recorded and transcribed by NT. Transcripts were de-identified and uploaded onto REDCap version 10, a web-based application used to capture data in a secure environment so that research teams can collect and store highly sensitive information ^23^ for data management and analysis. Data was coded systematically and manually by two researchers (NT and LMT) in Microsoft Word version 16. NT and LMT developed and iteratively refined the codebook beginning with *a priori* codes from the interview guide. We used the research questions to group the data and then look for similarities and differences. We developed a hierarchical coding framework to analyze texts based on participants feelings, opinions and experiences, with broader higher-order codes providing an overview and detailed lower order codes allowing for distinctions to be made within and between cases ^24^. NT and LMT each coded transcripts separately, discussed the codes, reconciled any discrepancies, and then summarised each code’s content. The codes were sorted into themes. These summaries formed the basis of our thematic content analysis. We achieved saturation from the 16 participants interviewed. No new codes occurred from the data, suggesting that no further or new information was being gathered or learned about the volunteers’ perceptions and experiences.

### Ethical approval

The EHPC study, consent forms and interview guides were approved by the National Health Sciences Research Committee (NHSRC) ethics committee (protocol number 19/08/2246) and the Liverpool School of Tropical Medicine (LSTM) ethics committee (protocol number 19-017). Participants were provided with an information sheet and verbal explanations, and they provided oral and written consent.

## Results

We interviewed 16 participants that were recruited between December 2019 and March 2020. Of the 16 participants, 15 were interviewed after all study procedures and after the final study visit and one participant was interviewed on a subsequent date rescheduled for participant convenience. The sample of participants is displayed in Table 2.

**Table 2.** Study participant demographics.

Gender	Age	Year of study
Male	23	4 ^th^ year of a degree programme
Male	25	4 ^th^ year of a degree programme
Male	22	4 ^th^ year of the degree programme
Male	20	1 ^st^ year of a degree programme
Male	26	4 ^th^ year of a degree programme
Female	18	2 ^nd^ year of a degree programme
Male	23	3 ^rd^ year of a degree programme
Male	24	3 ^rd^ year of a degree programme
Male	26	4 ^th^ year of a degree programme
Female	20	3 ^rd^ year of a degree programme
Male	27	4 ^th^ year of a degree programme
Male	25	4 ^th^ year of a degree programme
Male	27	4 ^th^ year of a degree programme
Female	20	3 ^rd^ year of a degree programme
Male	22	Completed Form 4, Malawi School Certificate of Education (MSCE)
Female	24	3 ^rd^ year of a degree programme

As displayed in Table 2, most of our participants were male (n=11), and the remaining were female (n=5). Most were 3rd and 4th-year university students, while 1 participant had completed their secondary school education (O-level equivalent). All participants were 18 years or above, and all indicated that this was the first time they had ever participated in a clinical research study.

We present our findings under four main themes. First, we present participants’ decision making around participation in the pneumococcal feasibility study, including their views on the public engagement event at the Polytechnic campus and study consenting procedures described above in the recruitment procedures section, and motivations for participating. Second, we describe their experiences with study procedures and methods, including safety monitoring plans and the three-night hotel stay. Third, we discuss views on compensation. Finally, we present the participants’ suggestions for engagement and recruitment for future CHIM.

### Participants’ decision-making and motivations for participating in the EHPC feasibility study

Most participants informed us that they decided to join the study after attending a public engagement event organised by the MLW research team.

“…the very first time they came to school [the Polytechnic], they presented the study, but there were some areas, which I did not understand, and there were questions, which I could not ask in the presence of others, you could like to ask in person with the team doing the research. I had that time to ask, and they told me all the relevant information about what is it all about for the research. So that helped me, that was a good move that I had that ample time to speak out anything I was afraid of, and they could answer me.” (CHIM 1028)

Participants particularly liked the private setting at the research clinic, which enabled them to ask questions they would not have done in their peers’ presence after the public engagement event and before enrollment.

Since most of the volunteers were students studying at a university, it was common for them to use the Internet to research the study independently. The participants informed us that they conducted Internet searches on the pneumococcal carriage model from the LSTM to understand the pneumococci bacteria they would be inoculated with and the inoculation procedures to assess the study risks.

“To know more about bacteria, I researched...I just Googled pneumococci, and the Internet explained what this bacteria, pneumococci was... They said that this is mostly or is mostly in kids, if I am not wrong. Those affected most are children or those whose immunity is low, like people with HIV and AIDS and the elderly whose immunity system is low that bacteria can affect them, not healthy adults... I wanted to know more about this study… It helped me to have confidence enough that I join the study.” (CHIM 1036)

One participant consulted their family in the process of deciding to participate in the study. While the relatives did not offer a direct opinion, the participant felt that this was their tacit way of giving permission and reassurance that a sensible decision was being made.

“Maybe the part I can explain about, which made me reach the point of deciding to participate was, it was when I came to explain to my relatives at home after I gave them the form after they read it through, I felt to say: ah I think I can participate (CHIM 1184).

The motivation to participate was varied and based on perceived individual benefits and societal value. As one participant put it,

“My interest is to focus much on research, so the most important reason is to participate and gain more knowledge” (CHIM 1069).

A couple of participants were motivated to participate as they were very keen to know their general health status, which included an HIV antigen test.

“I was so motivated when they said we would have your health check-up. In most cases, I have been looking for that, but then if you look at the money going to the hospital and then having the check-up, it would cost me a lot. So, I was partly motivated because of that that I should know how I am. Even though the study did not have that fully checking up of the entire body, you had just some special areas that you were supposed to look at, but still more it has been good” (CHIM1119).

Only one participant mentioned money as the primary motivating factor for participating in the EHPC study.

“Besides money [laughs], but otherwise no, I just wanted to take part… Anyway, okay, generally, money is a basic need, it is a necessity, so with sixty-seven thousand, it was at least an attractive package” (CHIM1010).

The social value of participating in the research was expressed in the form of altruism and patriotism. Almost all participants spoke of the desire to help humanity and Malawians impacted by pneumonia and the scientific community develop a better vaccine.

“I just felt like at least I should be one of the people that could contribute to something good to the entire nation because I know this could be. It is something that has been recommended in Malawi. Therefore, it would carry a certain value. So, I thought of being part of it” (CHIM 1119)

Patriotism, expressed in the form of self-sacrifice or selflessness, was articulated when one participant described himself as a “risk-taker”.

“Ah, here I can say that I had no worry considering that I am a risk-taker. So joining it was just like that. I think I made up my general mind that I am joining this, and if they are telling me that this is the way it is handled. You will experience this and that, and nothing else can harm me as they said that it worked 100% in Liverpool. I was, like ah. I think this is just okay after so my first days when I experienced nothing, I did not have a fear that anything can happen” (CHIM 1028)

While this participant expressed the view that he was a “risk-taker”, his motivation and ultimate decision to participate were balanced by assessing the risks and the potential benefits.

“My interest was that I would be the one involved in helping the community because I know that when a vaccine is found, and I was involved in the process of making the vaccine, you will be reaching to many people that I cannot personally reach” (CHIM 1028).

## Experiences with study procedures and methods

Participants described positive and negative aspects of the study procedures. Positive factors included staff attitudes, safety monitoring and support for health care.

Several participants commented on the research staff’s friendly nature, which helped participants feel comfortable with the study follow-up visits and clinical procedures.

“The nurses were friendly, the way they talked to us, the way they were handling us, it was just okay, and I could feel that sense of closeness, sure. I think, for me, I can say it was just okay, because even they were flexible may be telling them that we will meet at such a time or that maybe you have changed time due to some changes, they would understand. I think it is just okay” (CHIM, 1028).

Participants also appreciated the safety monitoring procedures, including the hotel accommodation for three nights after receiving the inoculum. The Grace Bandawe Conference Centre [GBCC] is situated 3.4 km (four-minute drive) from a private medical facility ( Mwaiwathu Private Hospital) to provide study participants with medical care should they experience a severe adverse event.

“My experience was great. Yeah, everything about food, the place that we were sleeping, and it was a comfortable place and a very conducive environment for research like this” (CHIM 1085).
“You provided a person with all the options which he or she can follow if at all is feeling unwell. For example, you have given us an allowance, let’s find somebody gets sick while he is maybe somewhere very far, so he could travel using that money to go to the hospital. You also gave us a card whereby you can go to the Mwaiwathu hospital at any time where we were feeling unwell. The third was being accommodated at Grace Bandawe, which is very close to the Mwaiwathu hospital. Now, that thing itself is very good and has caused my experience to be excellent as well” (CHIM 1143).

Participants also appreciated receiving other forms of immediate medical care and support as part of the study safety monitoring. This included being escorted by a nurse/fieldworker for the nights spent at the hotel, the ability to access the medical team at all hours via cellphone, and a safety information sheet listing possible side-effects, such as a temperature of >37.5°C, shivering, headache, new rash, drowsiness, cough, earache, and or new eye infection. Participants were also provided with a thermometer to monitor their temperature personally and provided antibiotics to take if their temperature was too high.

Because having a thermometer that’s, because one of the symptoms of you know these bacteria is a rise in body temperature. So, having that in mind, I think that is enough. Furthermore, that card, like the information sheet, was good. There were just so many points that you cannot miss out on them, you see, maybe you are feeling something, it was listed on the card, so, that was enough for me” (CHIM 1010).

There were mixed views on the actual inoculation procedures. Most participants expressed initial feelings of fear and apprehension about receiving the inoculum.

“At first, I was very afraid. Actually, I was so afraid of, okay, I that running nose, that’s exactly one thing that I hate the most. I fail to study when I have a runny nose, so I hate it a lot” (CHIM 1093).

Another participant also felt unwell after receiving the inoculum and reported experiencing fatigue.

“Somehow I was feeling like just tired, somehow a little headache and that general feeling that I am not well, but I cannot explain it the way it was, but just feeling that I am not okay” (CHIM 1051).

Other concerns about inoculation related to the potential harm that the bacteria can cause to others.

“Yes, you know the social world we live in we always associate with people. So whenever you are with a friend you have met, certain family and they have a kid, you feel like carrying the kid, but when you think of oh, I am going under this, so you are somehow refraining, avoiding meeting people, avoiding going to family members, like family friends to chat, like there are kids there they will need me to carry. I have a sister, and she has a kid and that day, the kid was seven months old, so I never held her. It was just like I was avoiding her (CHIM 1093).

But many also talked about feeling reassured because of the antibiotics the study provided, the independent research they had done before joining the study and the fact they did not experience any untoward event.

“I was nervous. A bacteria is a bacteria; it is a microorganism. Sometimes it can decide to misbehave. There can be something that would alter the body, like the chemical reactions and the likes, so, I was afraid, of course, I was nervous. However, I knew, like from education-wise, we know what bacteria do, and there are treatments for bacteria. Yea, so, if anything, then take the antibiotics” (CHIM 1010).

Despite the worries, one study participant reported feeling at ease with receiving the inoculum.

“My experience to say that after inoculation as I said at first that I was like somehow worried but in the process, it was found that the worry disappeared because I did not experience anything unusual or that I was feeling sick. I was just okay the way I was even before joining the research. Hence I had the confidence that aah, I think these things are all right because the way they explained before the research that there is nothing harmful that can happen after inoculation, after being inoculated the bacteria there was like no harm that happened in line with what they said” (CHIM 1028).

Participants were also asked about their experiences with sample procedures, including the throat swab, nasal scraps, nasal washes, blood and saliva samples. Most participants were comfortable having their blood and saliva samples taken. However, many found the nasal scrapes, nasal washes and throat swabbing procedures uncomfortable. Holding water in the nose and then expelling it created discomfort. Similarly, having a throat swab sample taken was awkward because of the gag reflex.

## Compensation

Questions regarding the appropriate compensation levels for CHIM research in Malawi required evaluation. Participant views on the monetary payments they received varied. Some participants felt that the amount of compensation provided was reasonable.

“The study was not that demanding, and comparing the risk and the compensation that we are getting, it is fair, yes. So, the study was not demanding that much, and the clinic was just in a convenient place. It was easy to get to the clinic. So, mainly I would say the compensation was mainly for the airtime and the transport I would use to get to the clinic” (CHIM 1150)

However, others felt the compensation was inadequate compared to the perceived health issues that might appear after study participation is complete.

“What we are dealing with here is the human health of which if somebody has been inoculated with that bacteria, we never know some further reactions in their bodies that could happen even after the study maybe one year after now or two years from now. You know some things may happen after a very long period. Maybe this will have an adverse effect in the future. We never know. So, if we are to look at the health status of that participant, then the money is not enough” (CHIM 1119).

Another participant believed that monetary compensation for research burdens could not be equated with the value of life. Therefore, money would not be able to compensate for the loss of life.

“It is just a little money… I told you that it is about health hazard when you are making a decision you are sure that you are putting your life at risk, you never know what may come out of the study” (CHIM 1127).

Apart from monetary compensation, participants were asked to reflect on what other payment forms would be suitable to offer in CHIM. One notable view was to provide participants with health insurance to help compensate an individual should they fall ill during and long after the study is completed. Other ideas suggested were to offer participants risk allowances to cover unexpected and unpredicted costs, including t-shirts and certificates of appreciation to acknowledge their role and contribution to research.

### Suggestions for engagement and recruitment in future CHIM research in Malawi

Participants in the study offered a mixture of suggestions on using community and public engagement platforms to communicate and promote understanding about CHIM research, particularly if future studies plan to recruit individuals from the broader community who are not as educated or literate as university students. Key among the suggestions offered were participant advocates in the public and community engagement activities to provide first-hand testimonials about their experiences in participating in CHIM research.

“For them to understand, they need people who have participated in this study, not just telling them that you have to do this but the experienced one” (CHIM 1127).

Another participant echoed the above sentiment and explained how involving former participants as advocates would help dispel possible misconceptions about this type of research that deliberately infects an individual with a disease-causing agent.

“some people might have misconceptions about the study. So, to hear from somebody who has gone through the study, their experience might be a bit more, how I can put this? They might feel a bit more reassured that somebody who is like me in some sort went through the study and is testifying. So, I can put it that way that the study is not as bad as I think it is or it is not what I think that the study is all about. So, to say for participant advocacy groups are very important” (CHIM 1150).

Social media platforms such as WhatsApp was also proposed as a more effective way to reach people.

“I feel like the approach is good, but maybe you can also employ social media, especially WhatsApp, because nowadays people are so active on WhatsApp. Sometimes just posting posters around, most people do not like looking at posters around, maybe with our generation. However, when something circulates on WhatsApp, it is easy to reach people. So, maybe you can include the spreading of the message using WhatsApp” (CHIM 1135).

Other recommendations included offering study participants full-time residency for the entire study period and home visits as another way to bolster CHIM safety and monitoring procedures.

## Discussion

This is the first study in Malawi to examine participants’ perceptions and experiences of participating in a CHIM. Participants reported initial fears and concerns before they enrolled. Their anxiety levels dissipated after inoculation, primarily because of the strong communication with the nursing team and assurances by the study safety monitoring procedures, including a three-night residential stay at the GBCC after inoculation. From this, we can infer that CHIM research is acceptable in Malawi. However, participation in CHIM research rests on three essential conditions: motivation to participate, compensation and advocacy. In this section, we compare our findings to existing literature and map out recommendations for future studies.

Our findings on the factors influencing the decision and motivation to participate in the study included altruism, patriotism, financial benefits and medical health checks. This mirrors previously reported results in Malawi, where access to health care, monetary and material incentives given were the main reported reasons for participation in biomedical research ^25, 26^. Furthermore, the decision to participate was also influenced by the methods used for recruitment. For example, the public engagement event held at the Polytechnic college, where the Liverpool EHPC research was presented and demonstrated to be safe, helped establish integrity and trust. Moreover, the research team’s attitude, which was described as friendly and flexible during screening and enrolment, further helped influence participants’ decisions to participate because they gained trust in the research team.

However, these views and experiences, which informed decisions to participate, can be partly attributed to cognitive dissonance theory. Cognitive dissonance suggests that individuals tend to seek consistency among their cognitions (i.e., beliefs and opinions) that can influence behaviours and actions ^27^. In turn, these beliefs and views can help consolidate the perception that researchers have their best interests at heart ^28^. Cognitive dissonance tends to occur in situations where an individual must choose between two incompatible beliefs. For example, believing that research participation may have risks versus feeling that the researchers would not have suggested participation if the activity was risky ^28^. Participants in the MARVELS study may have decided to participate and stay in the study to counter their beliefs around risk because of the study’s material and monetary incentives. This kind of reasoned behaviour must be approached with some degree of caution, particularly for the future of CHIM research in Malawi, as it relates to evaluating whether participants are making an informed decision to participate in the study. Future research on decision-making in CHIM needs to interrogate whether participants understand the differences between the study’s goals and personal goals.

While financial compensation for participation in research was a motivational factor, for the most part, our findings show that the notions of altruism, patriotism, perceived individual benefits, societal value, and volunteerism were primary reasons. Altruism, patriotism, and volunteerism were important aspects of achieving informed consent. This showed us that participants appreciated the risks, benefits and burdens. The issue of risk-allowances and health insurance pointed to participants’ concern about how researchers would take care of unforeseen or unanticipated research-related illnesses. Similar problems were noted in a study done in India that reported on public perceptions of CHIM ^29^. The MARVELS feasibility study paid for no-fault insurance for participants to compensate for those who would sustain research-related injuries. This is a regulatory requirement for high-risk research in Malawi.

Nevertherless, risk-allowance and health insurance require some consideration, mainly these concerns expose CHIM researchers to additional ethical obligations beyond simply paying for time and inconvenience. It requires scientists, regulatory and ethics experts to consider paying for risk in medical research, thus incorporating compensating for actual or possible harm that may occur due to a study. In essence, Grimwald *et al.* propose a “payment for risk model” that involves paying for time, pain, inconvenience, and risks associated with participation. The payment for risk and pain would cover unforeseen impacts participating in CHIM on participants mental health and physical well-being ^30^. The payment for risks and pain would be similar to the type of compensation provided to those taking on risky jobs, especially when those jobs are a direct service to society ^30^. Risk payment supports the legal obligation of responsibility in research. It reflects the ethical commitment to establish justice and fairness in research. It serves as a reminder to CHIM researchers in LMICs like Malawi that “an injury to one is an injury to all” ^31^. The payment of risk model would help build public trust by demonstrating a commitment to the value of life, which is consistent with the duty of care, especially in CHIM research, where the possibility to cause harm could have severe implications for public support and willingness to participate. Providing participants with clinical trial insurance, health insurance, and risk-allowances can be interpreted as the researcher and participant working together to share the risks and burdens inherent in CHIM research.

Participant advocate groups (PAGs) for CHIM in Malawi could be an essential tool for community and public engagement. This study’s findings highlight an opportunity to establish PAGs to help researchers conduct CHIM with public awareness. PAG members would comprise previous research participants who volunteer their time, knowledge and experience in research. Their role would not be to convince people to join in CHIM or facilitate recruitment, but rather to learn about the public’s fears, concerns and expectations ^32^ and to respond to the questions and concerns that communities and the general public may raise, ideally before the implementation of the study and their participation. PAGs, because of their experience, would help dispel misconceptions, increase awareness of health and medical research and improve engagement between researchers and the public ^33^. Moreover, PAGs could be a means to engender the ethical conduct of CHIM research further. Like community advisory groups (CAGs), PAGs would also be the eyes and ears of researchers and the community and ^34^, in so doing, be able to protect the public’s interest and help them gain trust in the research process ^33^.

Our study has some limitations. Before starting the MARVELS study, we did not collect qualitative data on initial feelings and questions or concerns that participants may have had before joining the research. This is because we anticipated that potential participants who chose to enrol in the study would be open about their opinions at the end of their participation in the study. However, future qualitative research work must consider including this approach to help deepen our understanding of the underlying motivation to participate in CHIM research in Malawi. Moreover, the participants were interviewed at the clinical research institution after exiting the study. This may have affected openness because of the perceived links between the interviewers and the research team. Also, we may have ignored others’ views; for a more rounded assessment on the experiences, additional insights on experiences and acceptability with frontline research staff would have been helpful. Wider opinions from family members would also be necessary for understanding acceptability beyond immediate participants’ views. This would have given us a better understanding of informed consent, acceptable levels of risk and compensation. A varied view of perspectives and experiences in implementing and participating in CHIM research will help deepen our understanding of acceptability in Malawi and contribute to the growing discourse and literature on the ethically acceptable approaches to CHIM, and develop ethical frameworks, guidelines and principles on how to conduct CHIM in LMICs.

## Conclusion

Though participants were, at first afraid of participating in the first CHIM human pneumococcal carriage research, participants’ experiences of CHIM in Malawi provide the basis of what can be acceptable and areas where study procedures could be adjusted. MARVELS will use these to design the next steps of their research. Additionally, findings will also inform regulatory thinking on guidelines, frameworks and principles on how ethics review committees in Malawi should handle the governance of CHIMs. Finally, future research ought to consider issues around the theory of cognitive dissonance and the role that it can play in explaining participant experiences and decision-making to bring greater transparency about the acceptability of CHIM in LMICs.

## Data availability

### Underlying data

The data generated and analysed are not publicly available because consent was not obtained for these to be made public even if anonymized but are available from the corresponding author on reasonable request.

## MARVELS consortium

- Clinical team: Clara Ngoliwa, Edward Mangani, Modesta Reuben and Helen Thomson
- Laboratory team: Chris Mkandawire, Simon Sichone, Raphael Kamng’ona, Mphatso Mayuni and Percy Mwenechanya
- Data managers: Joel Gondwe and Clemens Masesa
- Administration: Sandra Antoine
- Collaborators: Kate Gooding, Markus Gmeiner, Mike Parker, Andrew Pollard, Rob Heyderman, Jason Hinds, Mark Alderson, Chris Bailey, Marien de Jonge, Robert Kneller, Jeremy Brown, Jane Mallewa, David Goldblatt, Richard Malley, Jeff Weiser, Jonathon Grigg, Henry Mwandumba, and Debby Bogaert
