# Supplementary material for: “At first, I was very afraid”—a qualitative description of participants’ views and experiences in the first Human Infection Study in Malawi
Source: Wellcome Open Res. 2021 Oct 4;6:89. Originally published 2021 Apr 22. [Version 2] doi: 10.12688/wellcomeopenres.16587.2 (PMC8825950; doi:10.12688/wellcomeopenres.16587.2)
Supplement: Peer review history

# Peer review: Reviewer response for version 2

**DOI:** 10.21956/wellcomeopenres.18890.r46266

**Published:** 2021-10-12

**Authors:** Evelyne Kestelyn

**Affiliations:** 1 Oxford University Clinical Research Unit, Ho Chi Minh City, Vietnam

I have no further comments. The authors addressed all the comments thoroughly.

Is the work clearly and accurately presented and does it cite the current literature?

Yes

If applicable, is the statistical analysis and its interpretation appropriate?

Not applicable

Are all the source data underlying the results available to ensure full reproducibility?

Partly

Is the study design appropriate and is the work technically sound?

Yes

Are the conclusions drawn adequately supported by the results?

Partly

Are sufficient details of methods and analysis provided to allow replication by others?

Yes

Reviewer Expertise:

Clinical trials, Infectious Diseases, Human challenge ethical and governance frameworks

I confirm that I have read this submission and believe that I have an appropriate level of expertise to confirm that it is of an acceptable scientific standard.

# Peer review: Reviewer response for version 1

**DOI:** 10.21956/wellcomeopenres.18279.r43585

**Published:** 2021-05-25

**Authors:** Evelyn Muleba Kunda-Ng'andu

**Affiliations:** 1 Research Department, The Centre for Infectious Disease Research in Zambia, Lusaka, Zambia

Toto *et al.* explored the experiences, motivations and concerns of HIS participants in Malawi. The reasons for participation included altruism, patriotism, monetary and material incentives. Some participants expressed concerns about the risks involved vis-a-vi the value of compensation they received from the study. Participants recommended the use of advocate groups to engage the wider community.

 This article provides very relevant and timely information on HIS for those planning to undertake HIS studies, especially in LMICs. This is because understanding the experiences of actual HIS participants provides insights that may be applied for better results in a HIS in similar settings. The paper makes interesting reading and provides clear methods and steps used making it appropriate for replication.

 Just a few notes for the authors.

**Abbreviations**

 It is important for the authors to be consistent in the presentation of these. Some have been presented in lower case while others have been presented by Capitalizing each word.

**Quotes**

 Authors should re-look some of the quotes to ensure that they read clearly to reflect the theme in the discussion.

 The quote below was repeated on page 9 of the paper. It is presented on page 9 and repeated immediately on page 9. Please try and find another quote to replace ‘it’, on either page or better still delete it from page 9.

 “At first, I was very afraid. Actually, I was so afraid of,

 okay, I that running nose, that’s exactly one thing that

 I hate the most. I fail to study when I have a runny nose,

 so I hate it a lot” (CHIM 1093).

**Abstract/Reporting qualitative findings/Discussion**

 Authors should avoid the use of the word ‘likelihood’, as it is used to show probability. The first sentence of the results in the abstract should be revised to sound more qualitatively appropriate and clear.

**Introduction**

 Where the authors describe the HIS situation in LMICs, it would be imperative to note that countries like Zambia conducted a pilot HIS ^1^ and have since begun conduction a full study. Furthermore, South Africa has also started conducting a HIS.

**Aim of the study**

 From the paper, I gather that the aim of the research was to explore and describe the experiences; motivations and concerns of enrolled HIS participants. In the section of the aim of the study, authors say they aimed ‘to **assess acceptability** among the healthy adult volunteers who had completed the feasibility study, including their opinions on study recruitment and consent procedures, medical care and support, compensation, and community engagement’. There is a need to revise this for consistency.

**Discussion**

 The authors discuss the decision process using the theory of cognitive dissonance and that this could affect the behaviours and actions of the participants. This may have been implied in the statements provided, but the authors may consider discussing further that participants of ‘their’ study may have decided to participate and stay in the study as a counter reaction to the disbeliefs and conflicting feelings. The participants may have decided not to show this for various reasons including material and monetary incentives.

 I must state that it is a very important theory for consideration in a HIS as discussed in the paper. Well-done.

 Did the study have a psychologist to assess the mental health aspect of the study participants?

**Conclusion**

 The authors should consider expanding on the conclusion to state what they would suggest given what they found in the study; this may include for future research.

 Thank you for the opportunity to review this interesting piece.

Is the work clearly and accurately presented and does it cite the current literature?

Yes

If applicable, is the statistical analysis and its interpretation appropriate?

Not applicable

Are all the source data underlying the results available to ensure full reproducibility?

Yes

Is the study design appropriate and is the work technically sound?

Yes

Are the conclusions drawn adequately supported by the results?

Partly

Are sufficient details of methods and analysis provided to allow replication by others?

Yes

Reviewer Expertise:

Bioethics, Ethics of HIS, Public Health, Health Research, Qualitative research methods

I confirm that I have read this submission and believe that I have an appropriate level of expertise to confirm that it is of an acceptable scientific standard.

## References

[1] : A pilot study on use of live attenuated rotavirus vaccine (Rotarix™) as an infection challenge model. *Vaccine* .2020;38(46) : 10.1016/j.vaccine.2020.09.023 7357-7362 10.1016/j.vaccine.2020.09.023 33032844

# Response

**Published:** 2021-07-22

**Authors:** Neema Mtunthama Toto

23 ^rd^ June 2021

 To:          The Editors, Wellcome Open

 Re:         "At first I was very afraid" – A qualitative description of participants' views and experiences in the first Human Infection Study in Malawi [Version 1; peer review: 1 approved, 1 approved with reservations]

 Firstly, appreciate the encouraging words following the review of our manuscript. Second, we thank the reviewers for their significant observations and contributions to improving clarity in our findings' write-up. Below we present our point-by-point responses to the reviewers.

**Reviewer's Comments**

**Reviewer 1**

**Abbreviations **the authors need to be consistent in the presentation of these. For example, some have been presented in lower case, while others have been presented by Capitalizing each word.

**Authors' response**

 Thank you very much. We have correctly presented the abbreviations.

**Action taken**

 Please refer to the highlighted text on the abbreviations list on page 2

**Quotes **Authors should re-look some of the quotes to ensure that they read clearly to reflect the theme in the discussion.

 The quote below was repeated on page 9 of the paper. It is presented on page 9 and repeated immediately on page 9. Please try and find another quote to replace 'it' on either page or, better still, delete it from page 9.

 "At first, I was very afraid. Actually, I was so afraid of, okay, I that running nose, that's exactly one thing that

 I hate the most. I fail to study when I have a runny nose, so I hate it a lot" (CHIM 1093).

**Authors' response**

 Thanks for that helpful observation. We have removed redundant or irrelevant quotes throughout the manuscript results section and deleted the duplicate quote.

**Action taken**

 Please refer to the highlighted text on page 17.

**Abstract/Reporting qualitative findings/Discussion **Authors should avoid using the word 'likelihood', as it is used to show probability. The first sentence of the results in the abstract should be revised to sound more qualitatively appropriate and clear.

**Authors' response**

 Thank you very much. We have removed the word "likelihood" of participation altogether.

**Action taken**

 Please refer to the highlighted text on pages 1 and 21, respectively.

**Introduction **Where the authors describe the HIS situation in LMICs, it would be imperative to note that countries like Zambia conducted a pilot HIS and have since begun conduction a full study. Furthermore, South Africa has also started conducting a HIS.

**Authors' response**

 We have included information on Zambia and South Africa.

**Action taken**

 Please refer to the highlighted text on page 3.

**Aim of the study  **From the paper, I gather that the objective of the research was to explore and describe the experiences, motivations and concerns of enrolled HIS participants. In the section of the aim of the study, authors say they aimed 'to **assess acceptability **among the healthy adult volunteers who had completed the feasibility study, including their opinions on study recruitment and consent procedures, medical care and support, compensation, and community engagement. There is a need to revise this for consistency.

**Authors' response**

 Thank you very much. While the overall objective was to describe participants' experiences, motivations, and concerns enrolled in the study. Our specific objectives explore their opinions on study recruitment and consent procedures, medical care and support, compensation, and community engagement. This was done to establish some acceptability and inform the design of future HIS studies in Malawi. We hope that this rephrasing of our statements accurately conveys our objectives, specific aims and goals.

**Action taken**

 Please refer to the highlighted text on page 5 under the heading, Aim of the study.

**Discussion, **The authors, discuss the decision process using the theory of cognitive dissonance and that this could affect the behaviours and actions of the participants. This may have been implied in the statements provided. Still, the authors may consider discussing further that participants of 'their' study may have decided to participate and stay in the study as a counter-reaction to the disbeliefs and conflicting feelings. The participants may have decided not to show this for various reasons, including material and monetary incentives. I must state that it is a very important theory for consideration in a HIS, as discussed in the paper. Well-done.

**Authors' response**

 Thank you for your appreciation of the theory used to explain participant behaviours and actions. We have considered your suggestion on discussing the concept further.

**Action taken**

 Please refer to the highlighted text on page 22.

 Did the study have a psychologist to assess the mental health aspect of the study participants?

**Authors' response**

 No, the study did not conduct any psychometric tests on study participants. It was not an objective or included as part of the eligibility criteria.

**Action taken**

 N/A

**Conclusion **The authors should consider expanding on the conclusion to state what they would suggest given what they found in the study; this may include for future research.

**Authors' response**

 Thank you for your suggestion. We have included a statement on what future research may need to be considered to expand our understanding of cognitive dissonance and the role that it can play in explaining participant experiences and decision-making to bring greater transparency about the acceptability of HIS in LMICs.

**Action taken**

 Please refer to the highlighted text on page 24.

 We hope that we have appropriately and adequately addressed all the reviewer comments described above and highlighted (in yellow) in the revised manuscript.

 Please do not hesitate to contact me should you require further amendments.

 Kind regards,



 Lucinda Manda-Taylor, PhD

# Peer review: Reviewer response for version 1

**DOI:** 10.21956/wellcomeopenres.18279.r43583

**Published:** 2021-05-17

**Authors:** Evelyne Kestelyn

**Affiliations:** 1 Oxford University Clinical Research Unit, Ho Chi Minh City, Vietnam

Thank you for the opportunity to review this manuscript. This is clear and well written, contributing to the growing field of knowledge about the conduct of CHIM in LMICs.

**Introduction:**

 It would be beneficial to define from the start the study population not only as healthy adult volunteers but as students. I assume the literacy level but potentially also the socio-demographic status of these students might not be fully representative for the population in Malawi.

 Paragraph 2, In the text: '...there are ethical issues concerning HIS, **especially** in LMICs' Some authors argue the ethical issues are not very different across settings. See: Kaewkungwal J, Adams P, Sattabongkot J, Lie RK, Wendler D (2019) Conducting human challenge studies in LMICs: A survey of researchers and ethics committee members in Thailand. PLoS ONE 14(10): e0223619. ^1^ *'results suggest that, in the view of important stakeholders in Thailand, the ethical issues raised by human challenge studies in LMICS do not differ significantly from the ethical issues raised by human challenge studies in high income countries'. *I know this is contested but I would nuance this statement or provide further argumentation/references.

**Interviews:**

 The interview guide was piloted on two health workers. Again I assume health workers might have a better grasp of medical terminology or research terminology than the general population. Did you consider piloting this guide with the students?

 The interview guide mentions:'How does that compare to how you feel about it now?', "it" referring to decision making or general experiences? My main issue here is that the interviews are all exit interviews and that issues such as desirability or recall bias are not addressed. Most research shows a difference between initial worries and doubts about a study intervention and experiences after study completion where these feelings of worry fade away as the participants get more familiarized with the study staff and experience positive situations. Did you collect any data before the study start on initial feelings, questions?

 Experience with study procedures and methods.

 The quote about the runny nose is used twice, are there other quotes that you might use instead?

**Compensation:**

 I would encourage the authors to refer to the literature around compensation for harm. The idea of compensation after study completion or to pay for insurance is interesting. Given the current debate around payment for harm versus payment for risk in CHIM, this is certainly worth elaborating on. See:  

 Grimwade O, Savulescu J, Giubilini A, *et al.*

 Payment in challenge studies: ethics, attitudes and a new payment for risk model. Journal of Medical Ethics 2020;46:815-826. ^2^

**Discussion:**

 The discussion mentions initial fears dissipating but again this is at study end and it would have been very interesting to have initial (pre-enrolment data) versus end of study data.

 Regarding wider opinions; not only family members etc... could give valuable input but were any screen-failures or students who refused to participate interviewed?

Is the work clearly and accurately presented and does it cite the current literature?

Yes

If applicable, is the statistical analysis and its interpretation appropriate?

Not applicable

Are all the source data underlying the results available to ensure full reproducibility?

Partly

Is the study design appropriate and is the work technically sound?

Yes

Are the conclusions drawn adequately supported by the results?

Partly

Are sufficient details of methods and analysis provided to allow replication by others?

Yes

Reviewer Expertise:

Clinical trials, Infectious Diseases, Human challenge ethical and governance frameworks

I confirm that I have read this submission and believe that I have an appropriate level of expertise to confirm that it is of an acceptable scientific standard, however I have significant reservations, as outlined above.

## References

[1] : Conducting human challenge studies in LMICs: A survey of researchers and ethics committee members in Thailand. *PLoS One* .2019;14(10) : 10.1371/journal.pone.0223619 e0223619 10.1371/journal.pone.0223619 31600282PMC6786649

[2] : Payment in challenge studies: ethics, attitudes and a new payment for risk model. *J Med Ethics* .46(12) : 10.1136/medethics-2020-106438 815-826 10.1136/medethics-2020-106438 32978306PMC7719900

# Response

**Published:** 2021-07-22

**Authors:** Neema Mtunthama Toto

23 ^rd^ June 2021

 To:          The Editors, Wellcome Open

 Re:         "At first I was very afraid" – A qualitative description of participants' views and experiences in the first Human Infection Study in Malawi [Version 1; peer review: 1 approved, 1 approved with reservations]

 Firstly, appreciate the encouraging words following the review of our manuscript. Second, we thank the reviewers for their significant observations and contributions to improving clarity in our findings' write-up. Below we present our point-by-point responses to the reviewers.

**Reviewer's Comments**

**Reviewer 2**

**Authors' response**

**Action taken**

**Introduction: **It would be beneficial to define from the start the study population not only as healthy adult volunteers but as students.

**Authors response**

 Thank you, we have added healthy adult student volunteers so that it remains clear that these characteristics formed part of the eligibility criteria and recruitment plan.

**Action taken**

 Please refer to the highlighted text in the abstract and on pages 2 and 5.

 I assume the literacy level, but potentially also the socio-demographic status of these students, might not be fully representative for the population in Malawi.

**Authors response**

 Yes, it is correct that the literacy level is not fully representative of the population in Malawi. However, their socio-demographic status may well represent the general population of Malawi because most students enrolled in public universities in Malawi are from backgrounds that are not necessarily affluent.

**Action taken**

 N/A

 Paragraph 2, In the text: '...there are ethical issues concerning HIS, **especially **in LMICs' Some authors argue the ethical issues are not very different across settings. See Kaewkungwal J, Adams P, Sattabongkot J, Lie RK, Wendler D (2019) Conducting human challenge studies in LMICs: A survey of researchers and ethics committee members in Thailand. PLoS ONE 14(10): e0223619.

*'results suggest that, in the view of important stakeholders in Thailand, the ethical issues raised by human challenge studies in LMICS do not differ significantly from the ethical issues raised by human challenge studies in high-income countries. *I know this is contested, but I would nuance this statement or provide further argumentation/references.

**Authors response**

 Thank you for this helpful suggestion. We have included this statement.

**Action taken**

 Please refer to the highlighted text on page 3.

**Interviews: **The interview guide was piloted on two health workers. Again I assume health workers might have a better grasp of medical terminology or research terminology than the general population. Did you consider piloting this guide with the students?

**Authors response**

 Thank you for your excellent questions. We wanted to pilot the interview guide on health workers first because we wanted to avoid any risk of misconception about the study, given that this was the first study we would conduct in Malawi. We decided to test the questions on a health worker to ensure clarity and establish some relevance. While a health worker may better grasp medical terminology, we wanted to a) establish relevancy in the questions we were asking and b) we needed to obtain feedback where questions were ambiguous. Besides, our questions were broad and general enough for University students to understand once we had established clarity and relevance.

**Action taken**

 N/A

 The interview guide mentions: 'How does that compare to how you feel about it now?', "it" referring to decision making or general experiences? My main issue here is that the interviews are all exit interviews and that issues such as desirability or recall bias are not addressed. Most research shows a difference between initial worries and doubts about a study intervention and experiences after study completion, where these feelings of worry fade away as the participants get more familiarized with the study staff and experience positive situations. Did you collect any data before the study start on initial feelings, questions?

**Authors response**

 The question, "How does that compare to how you feel about it now" was asking the participant to reflect on how they felt after joining the study to the point of exiting the study. Since the interviews took place immediately after the participant concluded all study procedures and had formally exited the trial, we believe that recall bias was not affected much. We also mention in the manuscript that as social scientists within the MARVELS consortium, we tried to be as reflexive and as neutral as possible during the data collection and analysis process by paying careful attention to our positionality. We also ensured that where participants offered conflicting views, we probed around contradictory points during questioning in an encouraging and supportive way.

**Action taken**

 N/A because on page 7 of the manuscript, under the heading "Study site", we did mention how we carefully considered our position.

**Authors response**

 Finally, we did not collect any data before starting the study on initial feelings and questions. This is because we felt that participants would be open enough with their opinions even at the end of the study. However, it is a valid point that you have raised. We have noted it as a possible limitation of our research to contextualize our understanding of the underlying motivation to participate in the first HIS study in Malawi.

**Action taken**

 Please refer to the highlighted text on page 23.

 Experience with study procedures and methods.

 The quote about the runny nose is used twice, are there other quotes that you might use instead?

**Authors response**

 Thank you for noting this. We have addressed the concern by deleting the duplicate quote. We have opted not to include another quote because other participants echoed similar experiences.

**Action taken**

 Please refer to the highlighted text on page 17.

**Compensation: **I would encourage the authors to refer to the literature around compensation for harm. The idea of compensation after study completion or to pay for insurance is interesting. Given the current debate around payment for harm versus payment for risk in CHIM, this is certainly worth elaborating on. See:

 Grimwade O, Savulescu J, Giubilini A, *et al.*

 Payment in challenge studies: ethics, attitudes and a new payment for risk model. Journal of Medical Ethics 2020;46:815-826.

**Authors response**

 Thank you for pointing us to this important piece of literature. We have included the idea of payment for risk as part of the overall discourse on compensation in HIS research.

**Action taken**

 Please refer to page the highlighted text on page 23.

**Discussion: **The discussion mentions initial fears dissipating but again, this is at the study end, and it would have been very interesting to have initial (pre-enrolment data) versus the end of study data.

**Authors response**

 Thank you for your observation. Indeed, it would have been helpful to conduct pre-and post-interviews, and future qualitative research on participant opinions about HIS should consider this. We have taken note of this point and included it as a recommendation for future research

**Action taken**

 Please refer to the highlighted text on page 23.

 Regarding broader opinions, not only family members, etc., could give valuable input, but were any screen failures or students who refused to participate interviewed?

**References**

 1. Kaewkungwal J, Adams P, Sattabongkot J, Lie RK, et al.: Conducting human challenge studies in LMICs: A survey of researchers and ethics committee members in Thailand. *PLoS One*. 2019; **14 **(10): e0223619 PubMed Abstract | Publisher Full Text

 2. Grimwade O, Savulescu J, Giubilini A, Oakley J, et al.: Payment in challenge studies: ethics, attitudes and a new payment for risk model. *J Med Ethics*. **46 **(12): 815-826 PubMed Abstract | Publisher Full Text

 .

**Authors response**

 As with any clinical trial protocol, the eligibility and exclusion criteria is clearly defined. For the feasibility study, those participants with the natural serotype identified 6B were not included in the study. We only had one participant who was excluded based on these criteria. We did not experience any refusals from students who we invited to participate in the exit interview.

**Action taken**

 N/A

 We hope that we have appropriately and adequately addressed all the reviewer comments described above and highlighted (in yellow) in the revised manuscript.

 Please do not hesitate to contact me should you require further amendments.

 Kind regards,



 Lucinda Manda-Taylor, PhD
